# Development of the Children and Adolescents Physical Activity and Sedentary Questionnaire (CAPAS-Q): Psychometric Validity and Clinical Interpretation

**DOI:** 10.3390/ijerph192113782

**Published:** 2022-10-23

**Authors:** Alicia Fillon, Bruno Pereira, Jérémy Vanhelst, Joanna Baran, Julie Masurier, Terry Guirado, Yves Boirie, Martine Duclos, Valérie Julian, David Thivel

**Affiliations:** 1 Laboratory of the Metabolic Adaptations to Exercise under Physiological and Pathological Conditions (AME2P), EA 3533, Clermont Auvergne University, CRNH Auvergne, 63178 Clermont-Ferrand, France; 2 Biostatistics Unit (DRCI), Clermont-Ferrand University Hospital, 63000 Clermont-Ferrand, France; 3 Sorbonne Paris Nord University, Inserm U1153, Inrae U1125, Cnam, Nutritional Epidemiology Research Team (EREN), Centre of Research in Epidemiology and Statistics—University of Paris Cité (CRESS), 93017 Bobigny, France; 4 Institute of Health Sciences, Medical College, University of Rzeszow, 35-310 Rzeszow, Poland; 5 UGECAM Nutrition Obesity Ambulatory Hospital,13 Rue Molière, 63000 Clermont-Ferrand, France; 6 CRNH-Auvergne, 63178 Clermont-Ferrand, France; 7 INRA, UMR 1019, 63000 Clermont-Ferrand, France; 8 Department of Human Nutrition, Clermont-Ferrand University Hospital, G. Montpied, 63000 Clermont-Ferrand, France; 9 UFR Medicine, University Clermont Auvergne, 28 Place Henri Dunant, 63000 Clermont-Ferrand, France; 10 National Observatory for Physical Activity and Sedentary Behaviors (ONAPS), UFR Medicine, University Clermont Auvergne, 28 Place Henri Dunant, 63000 Clermont-Ferrand, France; 11 Department of Sport Medicine and Functional Explorations, Clermont-Ferrand University Hospital, G. Montpied, 63000 Clermont-Ferrand, France

**Keywords:** physical activity, sedentary behaviors, children, adolescents, questionnaire

## Abstract

Background: Pediatricians’ clinical practice and health interventions in youths require instruments with adequate psychometric properties to assess physical activity (PA), sedentary behaviors (SB), and their subdomains. Objective: To assess the psychometric properties of the Children and Adolescents Physical Activity and Sedentary-Questionnaire (CAPAS-Q) in healthy French children and adolescents. Methods: The CAPAS-Q has been developed through a rigorous collective procedure and consists of a 31 items self-administered questionnaire evaluating children’s 7-day PA and SB dimensions and subdomains. Participants (*n* = 103, aged 8–18 years old) completed the questionnaire twice (7 days apart) and wore an ActiGraph GT3X + accelerometer for 7 days. Anthropometric measurements and body composition were assessed. Results: Cronbach alpha for PA and SB dimensions were 0.71 and 0.68, respectively. Reproducibility was found moderate to good, with Lin’s coefficients of 0.69 and 0.68 for PA and SB dimensions, respectively. Reproducibility was higher for adolescents compared to children. PA dimension was positively correlated with moderate PA, vigorous PA, moderate to vigorous PA, and total PA times and negatively correlated with SB time (*p* < 0.05). SB dimension and screen time were positively correlated with SB time and negatively correlated with LPA, MPA, MVPA, and total PA times (*p* < 0.05). Spearman correlation coefficients were fair to moderate, ranging between 0.23–0.45. Conclusion: The CAPAS-Q proposes a reliable and valid evaluation of French children and adolescents’ PA and SB, providing clinicians with potential intervention levels to improve youth movement behaviors.

## 1. Introduction

Physical activity (PA) is positively associated with greater physical, mental, and social health in children and adolescents [1,2,3,4,5]. However, one can be very active while also engaging in high amounts of daily sedentary behaviors (SB) (i.e., any waking behavior in a sitting, reclining, or lying posture) [6]. While strong evidence in adults showed that PA attenuated without eliminating the cardiometabolic risk associated with high SB [7], pediatric studies of the last decade demonstrated that decreasing SB improved both physical and psychosocial health in youth [8,9,10,11,12,13]. Increasing PA and reducing SB would have independent and synergic effects on major health outcomes [13,14,15,16,17,18,19]. Thus, the World Health Organization (WHO) currently recommends at least 60 min of moderate to vigorous PA (MVPA) per day while minimizing SB, in particular screen time, to be less than 2 h per day for children and adolescents [20]. These recommendations are shared by most American and European countries, including France [21].

Assessing PA and SB in youths is therefore crucial for pediatrician’s clinical practice targeting at-risk patients as well as for public health interventions promoting movements-related behaviors. It remains, however, challenging. Indeed, while providing the most objective measurements, accelerometers have several economic and logistic limitations, are difficult to implement in large-scale studies, and do not provide information regarding the context of PA or SB [22,23,24]. Questionnaires are less accurate (due to their self-reported nature), remain language-specific and heterogeneous (as they are often validated only in specific aged or gender sub-groups) [25,26,27,28], and are rarely tested for both acceptable validity and reliability [28]. In their work, Saint-Maurice et al. underlined the large variability observed in the literature regarding the correlation between objectively measured MVPA (using accelerometers) and self-reported questionnaires, ranging from 0.19 to 0.75 [26]. On top of this important variability observed regarding physical activity, the actual literature clearly highlights the lack of reliable questionnaires to evaluate sedentary behaviors in youth. Camargo et al., for instance, only showed a coefficient of consistency for a sedentary time of 0.22 to 0.34 using a self-reported questionnaire in children [28]. Similarly, in their systematic review, Hidding et al. concluded an only fair to poor methodological validity of the questionnaire approaching sedentary behaviors in children [29].

Based on the importance of considering both physical activity and sedentary behaviors, there is, to our knowledge, a real need to develop easy-to-use, valid and reliable questionnaires assessing both PA and SB in children and adolescents [29]. For example, the most widely adopted French questionnaire to evaluate PA in children and adolescents has been developed for adults and remains surprisingly not validated in adults nor in children (i.e., Ricci et Gagnon) [30]. Effective and reliable questionnaires proposing an appropriate evaluation of both PA and SB are thus needed in children and adolescents. We, therefore, developed a brief questionnaire, the Children and Adolescents Physical Activity and Sedentary Questionnaire (CAPAS-Q), that proposes a simple and focused evaluation of children’s PA and SB dimensions. It considers, in particular, their context of practice (included in subdomains) and provides practitioners with a direct diagnosis of their potential levers of action.

The study aims to determine the internal validity and test-retest repeatability of the CAPAS-Q in healthy French children and adolescents aged 8 to 18 years old. A secondary aim is to assess the concurrent validity of CAPAS-Q using triaxial accelerometry.

## 2. Methods

### 2.1. Development of the Children and Adolescents Physical Activity and Sedentary Questionnaire (CAPAS-Q)

The CAPAS-Q (Supplementary Table S1) has been developed via the following process: (i) composition of an expert panel of scientific and clinical practitioners; including members of the National French Observatory for Physical Activity and Sedentary Behaviors (ONAPS, Clermont-Ferrand, France) (*n* = 3), the Pediatric rehabilitation Center UGECAM (*n* = 2), the Sport Sciences Faculties (*n* = 2) and university laboratories (*n* = 2). (ii) The collaborative work of this expert panel to identify the indicators needed in the clinical and preventive practices (overall, this stage took about 5 meetings organized over a 4-week period). (iii) The nomination of a working group of three members in charge of proposing appropriate questions and grading systems to assess these indicators. This sub-working group was composed of representatives of the clinical, scientific, and practical actors and met 4 times over a period of 2 weeks. (iv) Back-and-forth concertation’s between the expert panel members and the working group regarding the conception and phrasing of the questions. Questions were selected based on their scientific and clinical relevance, targeting the different dimensions of physical activity and sedentary behaviors in different contexts. (v) A collective decision regarding a first “preliminary” version of the CAPAS-Q (these two last steps, (iv) and (v) took about 3 weeks). (vi) The selection of a sample of 20 children and adolescents (and their parents) and realization of focus groups evaluating the understanding and usability of the questionnaire (4 focus groups were realized over a period of 5 weeks). (vii) The formalization of a revised version based on the results of the focus groups and confrontation with the 20 youths and their parents; (viii) validation of the final draft by the expert panel.

### 2.2. Description of the Children and Adolescents Physical Activity and Sedentary Questionnaire (CAPAS-Q)

The CAPAS-Q consists of a self-administered questionnaire containing 31 items developed to assess the 7-day PA and SB during a typical week. Importantly, this questionnaire has been developed to provide guidance to clinicians regarding PA and SB behaviors and not to precisely measure PA and SB times (e.g., in min per week). For each item (except question 15), responses are quoted from 1 to 4 for PA and from 1 to 6 for SB. The first 18 items were designed to assess the PA dimension, exploring its duration (i.e., the sum of items 1, 3, 6, 8, 10, 12, and 17) and intensity (i.e., the sum of items 2, 4, 7, 11, 13, and 18) within its different contexts of practice: school PA (i.e., the sum of 5 items from question 1 to question 5), non-school PA (i.e., the sum of 8 items from question 6 to question 14), and sports and leisure PA (i.e., the sum of 4 items from question 15 to question 18). Questions 16 to 18 were completed only when participants answered “yes” to item 15. The last 13 items were designed to assess the SB dimension, exploring whether it concerns screen (i.e., the sum of items 2, 6, and 7) or non-screen (i.e., the sum of items 1, 4, 5, 8, 9, 12 and 13) behaviors, the consecutive SB duration (i.e., the sum of items 3, 10 and 11) and the context of SB: school SB (i.e., the sum of 3 items from question 1 to question 3), non-school SB (i.e., the sum of 8 items from question 4 to question 11) and SB during transportation (i.e., the sum of items 12 and 13). 

The structure of the questionnaire was elaborated based on the example of the validated Physical Self-Description Questionnaire, proposing short questions with pre-determined answers. This structure has been mainly validated after the focus groups that provided feedback from the children and adolescents regarding the ability to fill in the questions and understand each item. The choice of the pre-determined answers was made based on the clinical experience and, once more, on the feedback of the participants.

### 2.3. Data Collection and Validation process

#### 2.3.1. Participants and Methods

A total of 120 children and adolescents aged between 8 and 18 years old took part in the study. These children and adolescents were recruited through advertisements sent to different academic, associative, cultural, and sports networks. Participants were classified according to age as children ((8–11) years old) or adolescents ((12–18) years old). The study was entirely detailed and explained to the participants and their legal representatives, and written informed consent was obtained. This study was conducted in accordance with the Helsinki declaration and was approved by the Ethics Committee CPP Sud Est VI (reference: 2020/CE 27).

After inclusion, participants were asked to complete the CAPAS-Q for the first time. Adolescents above 11 years old filled out the questionnaire themselves while children were assisted by their parents. Anthropometric characteristics were measured. Then, participants received verbal instructions concerning the use of accelerometers and were asked to wear them for 7 consecutive days, for a maximum amount of time (except while showering, bathing, or swimming). The accelerometers were positioned on their right hip. Seven days later, participants were asked to join the laboratory to fill in the questionnaire for the 2nd time, in the exact same conditions, and to give back the accelerometers. Importantly, the choice of a 7-day timeframe was made per available evidence showing good reliability of weekly recalls [31]. Indeed, the time between a questionnaire’s first and second administrations is known to influence the test-retest reliability, and the highest coefficients are usually obtained for a shorter timeframe [32].

#### 2.3.2. Anthropometric Measurements

Body weight and height were recorded to the nearest 0.1 kg and 0.5 cm, respectively, on participants wearing light clothes and bare-footed, using a digital scale (Seca, Les Mureaux, France) and a standard wall-mounted stadiometer (Seca, Les Mureaux, France). Body mass index (BMI) was calculated as weight (kg) divided by height squared (m^2^) and was plotted on sex- and age-specific French reference growth curves for the BMI percentile. Total fat mass was measured by bioelectrical impedance (Tanita MC780 multi-frequency) [33,34].

#### 2.3.3. Accelerometry Physical Activity and Sedentary Times

PA and SB were measured by an ActiGraph GT3X+ accelerometer (ActiGraph, Pensacola, FL, USA), previously used for validity and reliability studies in similar populations [32]. The Troiano et al. method has been used to identify the time that accelerometers were not worn: periods of 60 min (or more) of zero values were excluded [35]. Data were considered valid if the accelerometer was worn for at least 4 days (with at least 1 weekend day) and for at least 10 h per day between 8 a.m. and 10 p.m. each day. The sampling period was set to 5 epochs (100 Hz), and the outcome was expressed as minutes per day and percentage of wear time. Romanzini et al. cut-off points were used to translate acceleration counts into minutes per day of sedentary, light (LPA), moderate (MPA), and vigorous PA (VPA) [36]. MVPA was calculated as the sum of MPA and VPA. The ActiGraph data were downloaded using the software provided by the manufacturer (version 6.0, ActiGraph, Pensacola, FL, USA) and imported into SPSS v21 (IBM, Chicago, IL, USA) for data processing and screening. R package 4.0.2 accelerator (www.datahunter.es, accessed on 25 January 2022) was used to identify wear time between 8 a.m. and 10 p.m.

### 2.4. Statistical Analysis

Sample size estimation was fixed according to COSMIN recommendations [37]. Accordingly, it was decided to include a minimum of 100 participants in order to analyze the consistency and internal validity, reproducibility, and external validity with satisfactory statistical power. More precisely, rules-of-thumb for the number of subjects needed for internal consistency vary from 4 to 10 subjects per variable, with a minimum number of 100 subjects to ensure the stability of the variance-covariance matrix. For reproducibility, at least 50 patients were needed to highlight a positive rating for the reliability of at least 0.70. Accordingly, it was proposed to include 120 participants.

The statistical analyses used in this study were those usually used in studies to validate scales [38]. Continuous variables were presented as mean and standard deviation. In addition to descriptive statistics, the following psychometric properties of the CAPAS-Q scale were explored using: (i) Acceptability: data quality was considered satisfactory if more than 95% of the scale data were fully computable. Floor and ceiling effects were also calculated. (ii) Internal consistency was determined through Cronbach’s alpha coefficient (minimum accepted value: 0.70) and the item-rest correlation (accepted value: ≥0.30 and ≤0.70). (iii) For reproducibility, Lin’s concordance coefficient was used to determine the test-retest reliability for continuous outcomes. Values ≥ 0.70 were deemed satisfactory. (iv) Regarding convergent validity, relationships between dimensions and sub-domains of the CAPAS-Q and accelerometry parameters were studied using the correlation coefficient (Pearson or Spearman, according to statistical distribution). The results were interpreted according to the following rules of thumb [39]: <0.3: negligible correlation, 0.3–0.6: fair to moderate correlation, and >0.6: moderate to high correlation. Analyses were conducted for all participants and then in subgroups according to age and gender. Cronbach and Lin coefficients were compared between subgroups [40,41]. Statistical analyses were performed using Stata software (version 15, StataCorp., College Station, TX, USA). All statistical tests were carried out for a two-sided type I error at 5%. A Sidak’s type I error correction was applied to take into account two by two comparisons concerning correlation coefficients.

## 3. Results

**Figure 1:**
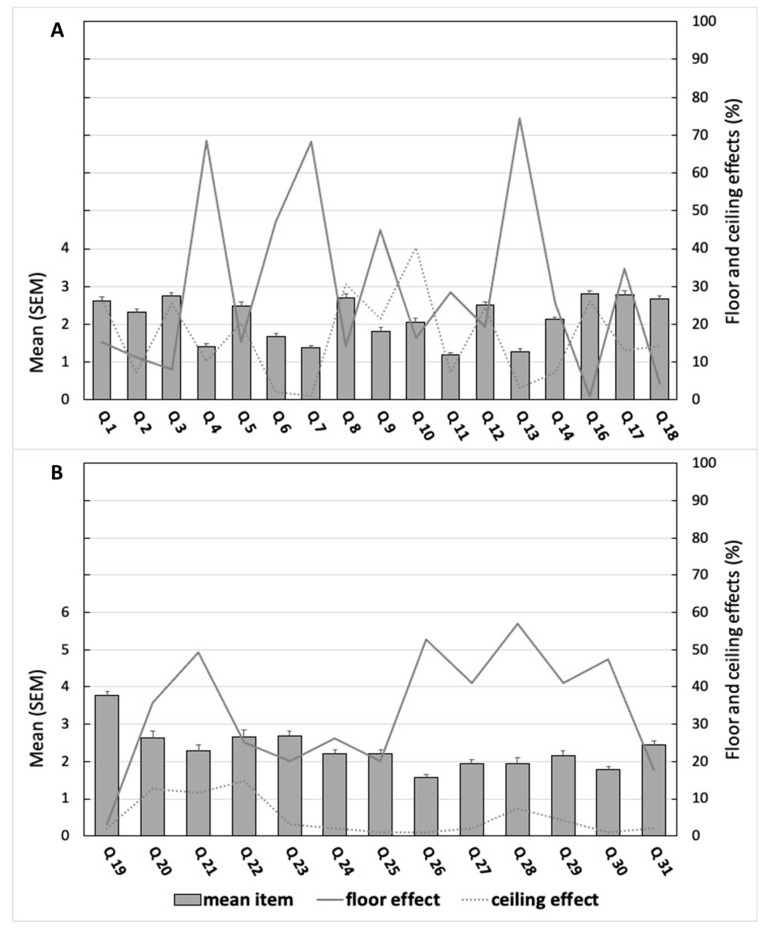
Acceptability of the CAPAS-Q for the overall sample (mean item ± SEM, floor and ceiling effects) for PA evaluation (**A**) and SB evaluation (**B**) dimensions.

Complete data were obtained for one hundred and three children and adolescents (mean age 12.2 ± 2.3 yrs, mean BMI 18.7 ± 2.9 kg·m^2^, 49.5% females, 62.1% adolescents). Anthropometric and accelerometry parameters are reported in Table 1. Females had a higher fat mass percentage (*p* < 0.001), a lower MVPA (*p* < 0.05), and total PA (*p* < 0.001) compared to males. Adolescents had a higher BMI (*p* < 0.001) and fat mass percentage (*p* < 0.05), and lower LPA (*p* < 0.01), MVPA (*p* < 0.05) and total PA (*p* < 0.001) compared to children. The acceptability of the CAPASQ, with floor and ceiling effects, is presented for both PA and SB dimensions in Figure 1.

### 3.1. Internal Consistency

**Figure 2:**
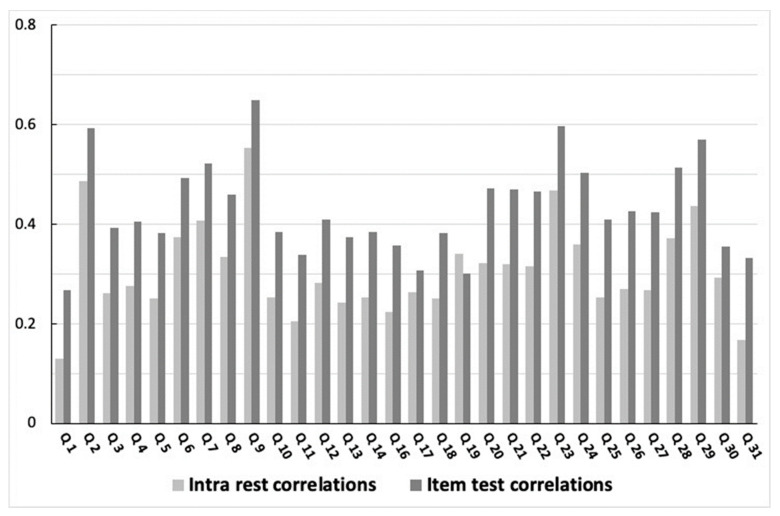
Item rest correlations and item test correlations of every question of the Children and Adolescents Physical Activity and Sedentary-Questionnaire (CAPAS-Q) for the overall sample.

The Cronbach alpha was 0.71 and 0.68 for PA and SB dimensions, respectively, for the overall sample. There was no difference between males and females (*p* = 0.659 and *p* = 0.278 for PA and SB dimensions, respectively) nor between children and adolescents (*p* = 0.254 and *p* = 0.32 for PA and SB dimensions, respectively). Item rest correlations and item test correlations for the overall sample are presented in Figure 2. No correlations between items were deemed too high (>0.8); however, correlations between a few items, especially item one (about number of hours of PA at school), and other items were somewhat low (<0.3). Item rest correlations and item test correlations for subsamples (males and females, children and adolescents) are presented in Supplementary Table S2.

### 3.2. Test-Retest Repeatability 

**Figure 3:**
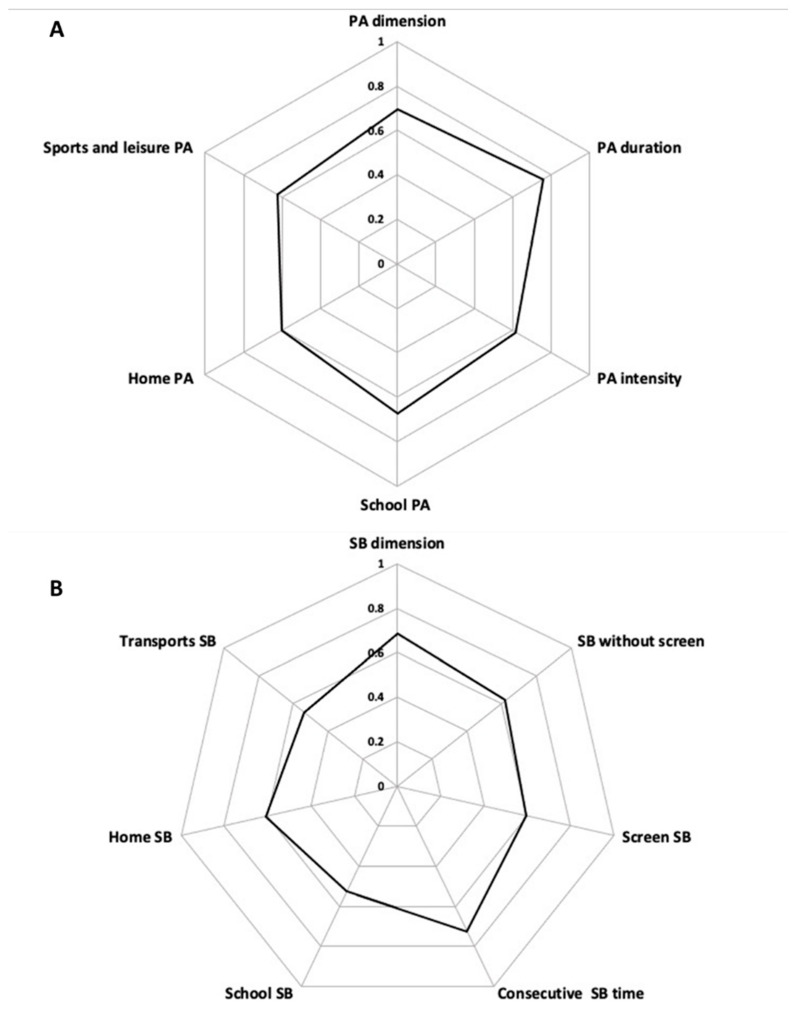
Lin’s coefficients of (**A**) physical activity (PA) and (**B**) sedentary behaviors (SB) dimensions and subdomains derived from the Children and Adolescents Physical Activity and Sedentary-Questionnaire (CAPAS-Q) for the overall sample.

**Figure 4:**
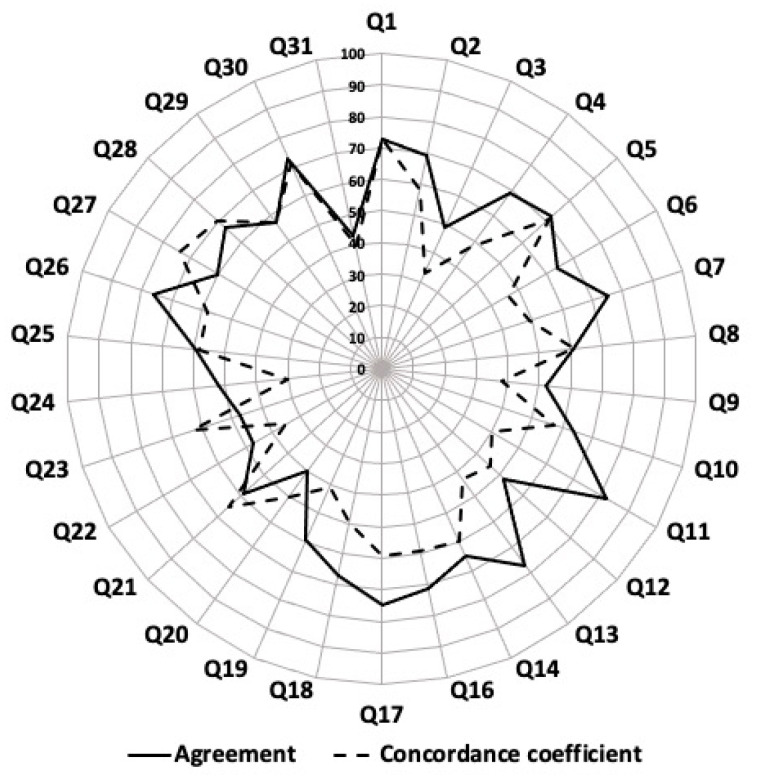
Agreement and concordant coefficient of every question of the Children and Adolescents Physical Activity and Sedentary-Questionnaire (CAPAS-Q) for the overall sample.

Lin’s coefficients are presented in Figure 3 for the PA and SB dimensions and their subdomains. They were no significant differences between females and males for both PA and SB dimensions (*p* = 0.193 and *p* = 0.076, respectively), nor between adolescents and children for the PA dimension (*p* = 0.667). However, Lin’s coefficient was higher in adolescents vs. children for the SB dimension (*p* < 0.01). Lin’s coefficients for subsamples are presented in Supplementary Table S3. Agreements and concordant coefficients for every question of the CAPAS-Q are presented in Figure 4.

### 3.3. Concurrent Validity

**Figure 5:**
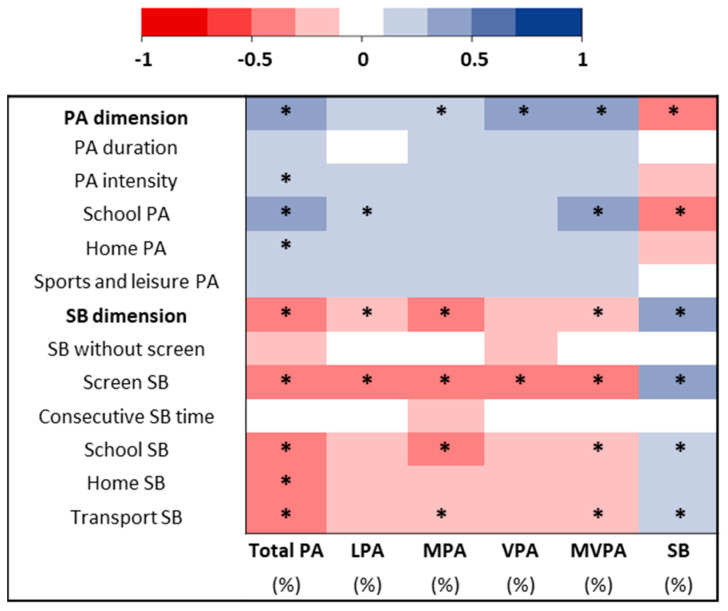
Heatmap representation of the correlations between physical activity (PA) and sedentary behaviors (SB) dimensions and subdomains derived from the Children and Adolescents Physical Activity and Sedentary-Questionnaire (CAPAS-Q) and accelerometry variables for the overall sample. The darkest is the box, and the higher is the correlation. * *p*-value < 0.05. LPA: light physical activity; MPA: moderate physical activity; MVPA: moderate to vigorous physical activity; PA: physical activity; VPA: vigorous physical activity; SB: sedentary behavior.

The correlation between PA and SB dimensions and subdomains and accelerometry parameters are presented in Figure 5. PA dimension was positively correlated with MPA, VPA, MVPA, and total PA (*p* < 0.05) times and negatively correlated with SB time (*p* < 0.05). SB dimension was positively correlated with SB time (*p* < 0.05) and negatively correlated with LPA, MPA, MVPA, and total PA times (*p* < 0.05). Screen SB was positively correlated with SB time and negatively correlated with LPA, MPA, MVPA, and total PA times (*p* < 0.05). School and transport SB subdomains were both positively correlated with SB time and negatively correlated with MPA, MVPA, and total PA times (*p* < 0.05).

## 4. Discussion

The present study proposes a newly developed brief self-reported questionnaire (the CAPAS-Q) specifically targeting, for the first time simultaneously, PA and SB, their durations, main contexts (e.g., school and non-school setting, institutional or non-institutional leisure activities and transports), and subdomains (e.g., duration and intensity for PA and screen, consecutive time for SB). Importantly, the CAPAS-Q has not been developed to precisely quantify PA and SB times but to provide a qualitative evaluation of these movement-related behaviors and their characteristics in children and adolescents. According to our results, the CAPAS-Q is a reliable and valid questionnaire whose dimensions correlate with device-based measures of youth movement behaviors.

Indeed, as underlined by the present analysis, the CAPAS-Q shows an acceptable internal consistency and a moderate to good test-retest reliability. Both PA and SB dimensions were found to correlate with accelerometry measurements. Importantly, although several systematic reviews showed generally modest validity and reliability of such questionnaires when assessing movement-related behaviors in youth [24,29,42], the Cronbach alpha of 0.71 for the PA dimension and of 0.68 for the SED dimension observed in the present work clearly highlights the highly acceptable validity of the CASP-Q. Furthermore, the test-retest reliability results were also fully concordant with what has been previously observed with other PA questionnaires used in young subjects within the same timeframe [43]. Although children were asked to complete the questionnaire with the help of their parents, lower repeatability has been shown in children compared to adolescents. The CAPAS-Q’s validity and reliability were not meaningfully different between boys and girls.

Otherwise, despite subjective decisions in data reduction (choice of cut-points for intensity levels, the minimum number of valid days and of valid hours per day, and the definition of non-wear time), the use of accelerometry is definitely a strength of the present study [24]. PA and SB dimensions, particularly screen habits, school, and transport SB subdomains, correlate with the accelerometry measurements, with Spearman correlations for concurrent validity ranging from fair to moderate, which is once again within the range reported for most of PA questionnaires in youths [31,42]. Correlations remain lower compared to the ONAPS Physical Activity Questionnaire assessing both PA and SB dimensions and recently validated in French adults [44].

The important point is that this questionnaire is easy to understand and to fill in for children, adolescents, and parents, as well as time-saving for clinicians and evaluators. Moreover, above all the statistical results underlying the satisfying validity and reliability of the CAPAS-Q, it really needs to be stressed that the CAPAS-Q, as a self-reported subjective tool, has been elaborated as a field and clinical tool to provide indicators regarding overall movement behaviors: it does not aim to quantify PA and SB times precisely but proposes an evaluation of PA and SB subdomains and settings, giving, in fine, practitioners some indications regarding their potential options and levers of actions to promote movement-related behaviors and reduce SB time. As an example, instead of providing a general SB time indication, the CAPAS-Q highlights whether the main SB time of the child is spent at home or outside, seated and/or in front of screens. This can then favor a better understanding of children and adolescents’ movement-related behaviors and help elaborate better preventive public health messages and strategies and better and more focused behavior change interventions. In that sense, it did not appear pertinent to propose overall and subdomain scores based on quantitative information. Either, it can be proposed to clinicians or practitioners to estimate the behavioral profile of children by scoring each main dimension (PA and SB) as well as their respective subdomains (duration, intensity, nature, and context) from 1 to 4 for PA and 1 to 6 for SB (calculating the means of their items). Then it can be proposed that whatever the domain or subdomain for PA, a child with a score from 1 to 2 presents a somehow unhealthy active behavior, from 2 to 3 a behavior that can be further improved, and a score of 4 presents a satisfying behavior. Similarly, for SB, a child with a score from 1 to 2 presents a satisfying related behavior; from 2 to 4, a behavior that can be further improved; and 4 and above, a behavior that needs to be improved.

These results obviously have to be interpreted in light of some limitations, and further research must be conducted. Although the total sample size remains satisfactory, a larger subsample for the age range or genders would have allowed more precise and detailed analyses. Indeed, the sample might certainly not be representative of the French children and adolescents population. Similarly, the inclusion of lean children and adolescents might restrict these results. Further studies are needed to assess the acceptability and reliability of the CAPAS-Q in specific populations, such as youth with overweight and obesity. Although the CAPAS-Q proposes a detailed evaluation of the children and adolescents’ movement behaviors and their sub-dimensions and domains, it does not provide information regarding their motivations and barriers to engaging in PA or SB. It also does not provide information regarding their preceding experiences, which is important information that should be considered in future versions of the questionnaire. Finally, although this was not the aim of the present work, the sensibility to changes in the CAPAS-Q should be explored in future studies.

## 5. Conclusions

The present work suggests the CAPAS-Q as a reliable and valid questionnaire to use in French children and adolescents. The development of the CAPAS-Q fills an important gap in youths’ movement-related behaviors and health research by providing pediatricians and researchers with a clinically relevant tool that demonstrates acceptable psychometric properties. By assessing PA and SB subdomains, the CAPAS-Q can help health professionals identify levers of individual improvement for their patients. 

## Figures and Tables

**Table 1:** Characteristics of the population.

	Overall (*n* = 103)	Males (*n* = 52)	Females (*n* = 51)	(8–11) Years Old (*n* = 39)	(12–18) Years Old (*n* = 64)
**Anthropometry**					
Age (year)	12.2 ± 2.3	11.8 ± 1.9	12.5 ± 2.5	9.5 ± 1.1	13.4 ± 1.4 ^###^
Height (cm)	155.5 ± 13.9	155.7 ± 14.6	155.2 ± 13.4	140.2 ± 9.5	161.4 ± 10.5 ^###^
Weight (kg)	46.3 ± 10.5	46.1 ± 14.8	46.5 ± 12.2	32.6 ± 5.4	51.4 ± 12.0 ^###^
BMI (kg·m^−2^)	18.7 ± 2.9	18.5 ± 3.1	18.9 ± 2.8	16.5 ± 1.8	19.6 ± 3.1 ^###^
Z-BMI (z-score)	−0.063 ± 0.94	−0.129 ± 1.13	0.002 ± 0.73	−0.10 ± 0.9	−0.04 ± 0.9
BMI (percentile)	48.5 ± 27.7	47.2 ± 30.8	49.8 ± 24.5	46.5 ± 26.8	49.5 ± 28.2
Fat-free mass (kg)	34.7 ± 9.7	35.7 ± 11.4	33.5 ± 7.6	24.9 ± 8.2	37.5 ± 10.2
Fat mass (%)	20.5 ± 4.7	17.9 ± 4.9	23.16 ± 4.4 ***	19.4 ± 3.2	21.6 ± 5.9 ^#^
**Accelerometry First Questionnaire**					
Total PA (min/day)	275 ± 56	298 ± 61	240 ± 51 ***	346 ± 57	268 ± 57 ^###^
LPA (min/day)	183 ± 37	196 ± 40	163 ± 33	227 ± 30	169 ± 40 ^##^
MPA (min/day)	46.6 ± 14.5	50.0 ± 14	41.7 ± 13.6	60 ± 15	45 ± 13 ^##^
VPA (min/day)	45 ± 21	51 ± 23	35 ± 19 **	59 ± 21	44 ± 23
MVPA (min/day)	92 ± 34	101 ± 34	76 ± 28 *	119 ± 35	89 ± 33 ^#^
Sedentary time (min/day)	532 ± 96	519 ± 80	551 ± 115	472 ± 62	541 ± 103 ^#^

BMI: body mass index; LPA: low physical activity, MPA: moderate physical activity; VPA: vigorous physical activity; MVPA: moderate to vigorous PA. Different from males: * *p* < 0.05; ** *p* < 0.01, *** *p* < 0.001. Different from age (8–11) years old: ^#^ *p* < 0.05; ^##^ *p* < 0.01, ^###^ *p* < 0.001.

## Data Availability

In the AME2P laboratory, without being publicly available.

## Supplementary Materials

The following are available online at https://www.mdpi.com/article/10.3390/ijerph192113782/s1. Table S1: the Children and Adolescents Physical Activity and Sedentary Questionnaire (CAPAS-Q). Table S2: Item rest correlations and item test correlations of every question of the Children and Adolescents Physical Activity and Sedentary-Questionnaire (CAPAS-Q) for the overall sample and subsamples (males and females, children and adolescents). Table S3: Lin’s coefficient of the PA and SB dimensions and subdomains derived from the Children and Adolescents Physical Activity and Sedentary-Questionnaire (CAPAS-Q) for the overall sample and subsamples (males and females, children and adolescents).
