# Supplementary material for: Development of the Children and Adolescents Physical Activity and Sedentary Questionnaire (CAPAS-Q): Psychometric Validity and Clinical Interpretation
Source: Int J Environ Res Public Health. 2022 Oct 23;19(21):13782. doi: 10.3390/ijerph192113782 (PMC9655272; doi:10.3390/ijerph192113782)
Supplement: Supplementary file 1 [file ijerph-19-13782-s001.zip › Supplementary Table S1 CAPAS-Q.pdf]

Nom/ Prénom de l'enfant : Date de naissance : Questionnaire renseigné le :

# CAPAS-Q

*Children and Adolescents Physical Activity en Sedentary Questionnaire*

# QUESTIONNAIRE SUR L'ACTIVITÉ PHYSIQUE ET LA SÉDENTARITÉ DE L'ENFANT DE 8 À 18 ANS

## A REMPLIR PAR L'ENFANT

**Le questionnaire est divisé en 2 parties :**

**- la première en lien avec l'activité physique**

**- la deuxième avec la sédentarité.**

Entoure les réponses qui reflètent le plus ce que tu fais.

**Une seule réponse est possible par question.**

Toutes les informations recueillies seront confidentielles.

**Il n'existe pas de bonnes ou de mauvaises réponses.**

Nom/ Prénom de l'enfant : Date de naissance : Questionnaire renseigné le :

CAPAS-Q (Fillon *et al.*, 2022)

# 1ÈRE PARTIE : ACTIVITÉS PHYSIQUES

**Les questions 1 à 5** concernent ton niveau d'activité physique lorsque tu es **dans ton établissement scolaire ou d'accueil la journée** (chez l'assistante maternelle, au centre de loisirs, à la garderie, à l'école, au collège, au lycée, etc.).

**Les questions 6 à 14** concernent ton niveau d'activité physique **en dehors de ton établissement scolaire ou d'accueil la journée**. Tu dois donc penser aux moments où tu es à la maison, chez des amis, de la famille, au parc, etc.

**Les questions 15 à 18** concernent ton niveau d'activité physique **pendant tes activités de loisirs, en club ou en association**.

**Il est très important que tu distingues ces trois parties, afin d'être le plus proche possible de tes comportements réels.**

## ETABLISSEMENT SCOLAIRE / D'ACCUEIL EN JOURNÉE

| | | | | |
|---|---|---|---|---|
| **Q1.** En moyenne, combien d'heures d'activité physique en cours d'EPS et à l'USEP/UNSS pratiques-tu **par semaine ?** | Moins de 2h | 2h à 4h | 4h à 6h | Plus de 6h |
| **Q2.** Généralement, pendant ces heures d'activité physique est-ce que tu transpires ou est-ce que tu es essoufflé(e) ? | Pas du tout | Un peu | Moyennement | Beaucoup |
| **Q3.** En moyenne, **chaque jour**, combien de temps passes-tu à marcher, à courir (en récréation, à la garderie, etc.) : | Moins de 15 minutes | 15 à 30 minutes | 30 minutes à 1h | Plus d'1h |
| **Q4.** Généralement, pendant ces temps de marche, est-ce que tu transpires ou est-ce que tu es essoufflé(e) ? | Pas du tout | Un peu | Moyennement | Beaucoup |
| **Q5.** En moyenne, combien d'étages montes-tu à pied **chaque jour** ? | Moins de 2 | 3 à 5 | 6 à 10 | Plus de 10 |

## HORS ÉTABLISSEMENT SCOLAIRE / D'ACCUEIL EN JOURNÉE

| | | | | |
|---|---|---|---|---|
| **Q6.** En moyenne, **les jours d'école**, quand tu rentres à la maison, combien de temps passes-tu **par jour** à jouer **(sans être assis(e)) ?** | Moins de 30 minutes | 30 minutes à 1h | 1h à 1h30 | Plus d'1h30 |
| **Q7.** Généralement, lors de ces temps de jeux, est-ce que tu transpires ou est-ce que tu es essoufflé(e) ? | Pas du tout | Un peu | Moyennement | Beaucoup |
| **Q8.** En moyenne, **les jours de week-end ou de vacances**, combien de temps passes-tu **par jour** à jouer (sans être assis(e)) ? | Moins de 30 minutes | 30 minutes à 1h | 1h à 2h | Plus de 2h |
| **Q9.** Généralement, lors de ces temps de jeux, est-ce que tu transpires ou est-ce que tu es essoufflé(e) ? | Pas du tout | Un peu | Moyennement | Beaucoup |
| **Q10.** En moyenne, **les jours d'école**, combien de temps **par jour** marches-tu (pour aller à l'école, pour te promener, pour aller voir tes amis, etc.) ? | Moins de 15 minutes | 15 à 30 minutes | 31 minutes à 1h | Plus d'1h |
| **Q11.** Généralement, lors de ces temps de marche, est-ce que tu transpires ou est-ce que tu es essoufflé(e) ? | Pas du tout | Un peu | Moyennement | Beaucoup |
| **Q12.** En moyenne, **les jours de week-end ou de vacances**, combien de temps **par jour** marches-tu (pour te promener, pour aller voir tes amis, etc.) ? | Moins de 15 minutes | 15 à 30 minutes | 31 minutes à 1h | Plus d'1h |
| **Q13.** Généralement, lors de ces temps de marche, est-ce que tu transpires ou est-ce que tu es essoufflé(e) ? | Pas du tout | Un peu | Moyennement | Beaucoup |
| **Q14.** Combien d'étages, en moyenne, montes-tu à pied **chaque jour** ? | Moins de 2 | De 3 à 5 | De 6 à 10 | Plus de 10 |

## ACTIVITÉS SPORTIVES ET DE LOISIRS

| | | | | |
|---|---|---|---|---|
| **Q15.** Pratiques-tu une ou plusieurs activités physiques ou de loisirs tous les mois (en dehors de l'école) ? Par exemple : jouer au football, au rugby, au basketball, danser, faire de la gymnastique, **dans un club ou dans une association.** *Si tu entoures la réponse « Non », tu peux passer à la question 19.* | Non | | | Oui |
| **Q16.** Combien de fois pratiques-tu l'ensemble de ces activités ? | 1 à 3 activités par mois | 1 à 2 activités par semaine | 3 activités par semaine | Plus de 3 activités par semaine |
| **Q17.** En moyenne, combien de temps durent **chacune des activités physiques** que tu pratiques en club ou en association ? | Moins de 45 minutes | 45 minutes à 1h30 | 1h30 à 2h15 | Plus de 2h15 |
| **Q18.** Quand tu pratiques ces activités, est-ce que tu transpires ou est-ce que tu es essoufflé(e) ? | Pas du tout | Un peu | Moyennement | Beaucoup |

# 2ÈME PARTIE : SÉDENTARITÉ

**Les questions 19 à 21** concernent ton niveau de sédentarité lorsque tu es dans ton établissement scolaire ou d'accueil la journée (chez l'assistante maternelle, au centre de loisirs, à la garderie, à l'école, au collège, au lycée, etc.).

**Les questions 22 à 29** concernent ton niveau de sédentarité en dehors de ton établissement scolaire ou d'accueil. Tu dois donc penser aux moments où tu es à la maison, chez des amis, de la famille, etc.

**Les questions 30 et 31** concernent tes comportements sédentaires pendant les temps de transport.

**Il est très important que tu distingues ces trois parties, afin d'être le plus proche possible de tes comportements réels.**

## ETABLISSEMENT SCOLAIRE / D'ACCUEIL EN JOURNÉE

| | | | | | | |
|---|---|---|---|---|---|---|
| **Q19.** En moyenne, combien de temps passes-tu assis(e) **par jour** en classe, à la récréation, à la cantine, etc. (***sans compter le temps passé devant un écran***) ? | Moins de 2h | 2h à 4h | 4h à 6h | 6h à 8h | 8h à 10h | Plus de 10h |
| **Q20.** En moyenne, combien de temps passes-tu devant un écran **par jour** en classe, à la récréation, à la cantine, etc. (ordinateur, tablette, téléphone, jeux vidéo, etc.) ? | Moins de 30 minutes | 30 minutes à 1h | 1h à 1h30 | 1h30 à 2h | 2h à 2h30 | Plus de 2h30 |
| **Q21.** En moyenne, combien de fois dans une journée restes-tu assis(e) plus d'1h30, sans bouger au moins 2 minutes d'affilée ? | 0 fois | 1 fois | 2 fois | 3 fois | 4 fois | Plus de 4 fois |

## HORS ÉTABLISSEMENT SCOLAIRE / D'ACCUEIL EN JOURNÉE

| | | | | | | |
|---|---|---|---|---|---|---|
| **Q22.** En moyenne, **les jours d'école**, combien de temps passes-tu assis(e) **par jour** (*sans compter : le temps passé devant un écran, à faire tes devoirs et à dormir*) ? | Moins de 30 minutes | 30 minutes à 1h | 1h à 1h30 | 1h30 à 2h | 2h à 2h30 | Plus de 2h30 |
| **Q23.** En moyenne, **les jours de week-end ou de vacances**, combien de temps passes-tu assis(e) **par jour** (*sans compter : le temps passé devant un écran, à faire tes devoirs et à dormir*) ? | Moins de 30 minutes | 30 minutes à 1h | 1h à 1h30 | 1h30 à 2h | 2h à 2h30 | Plus de 2h30 |
| **Q24.** En moyenne, **les jours d'école**, combien de temps passes-tu devant un écran **par jour** (ordinateur, tablette, téléphone, jeux vidéo, TV, etc.) ? | Moins d'1h | 1h à 2h | 2h à 3h | 3h à 4h | 4h à 5h | Plus de 5h |
| **Q25.** En moyenne, **les jours de week-end ou de vacances**, combien de temps passes-tu devant un écran **par jour** (ordinateur, tablette, téléphone, jeux vidéo, TV, etc.) ? | Moins de 2h | 2h à 4h | 4h à 6h | 6h à 8h | 8h à 10h | Plus de 10h |
| **Q26.** En moyenne, **les jours d'école**, combien de temps passes-tu à faire tes devoirs **par jour** ? | Moins d'1h | 1h à 2h | 2h à 3h | 3h à 4h | 4h à 5h | Plus de 5h |
| **Q27.** En moyenne, **les jours de week-end ou de vacances**, combien de temps passes-tu à faire tes devoirs **par jour** ? | Moins d'1h | 1h à 2h | 2h à 3h | 3h à 4h | 4h à 5h | Plus de 5h |
| **Q28.** **Les jours d'école**, combien de fois dans une journée restes-tu assis(e) plus d'1h30, sans bouger au moins 2 minutes d'affilée ? | 0 fois | 1 fois | 2 fois | 3 fois | 4 fois | Plus de 4 fois |
| **Q29.** **Les jours de week-end ou de vacances**, combien de fois dans une journée restes-tu assis(e) plus d'1h30 sans bouger au moins 2 minutes d'affilée ? | 0 fois | 1 fois | 2 fois | 3 fois | 4 fois | Plus de 4 fois |

## TRANSPORTS

| | | | | | | |
|---|---|---|---|---|---|---|
| **Q30.** En moyenne, **les jours d'école**, combien de temps passes-tu **par jour** en voiture, en bus, etc. (pour aller à l'école, au sport, aux courses, voir des amis, etc.) ? | Moins de 30 minutes | 30 minutes à 1h | 1h à 1h30 | 1h30 à 2h | 2h à 2h30 | Plus de 2h30 |
| **Q31.** En moyenne, **les jours de week-end ou de vacances**, combien de temps passes-tu **par jour** en voiture, en bus, etc. (pour aller au sport, aux courses, voir des amis, etc.) ? | Moins de 30 minutes | 30 minutes à 1h | 1h à 1h30 | 1h30 à 2h | 2h à 2h30 | Plus de 2h30 |

MERCI !
