# Supplementary material for: Development of the Children and Adolescents Physical Activity and Sedentary Questionnaire (CAPAS-Q): Psychometric Validity and Clinical Interpretation
Source: Int J Environ Res Public Health. 2022 Oct 23;19(21):13782. doi: 10.3390/ijerph192113782 (PMC9655272; doi:10.3390/ijerph192113782)
Supplement: Supplementary file 1 [file ijerph-19-13782-s001.zip › Supplementary Table S2.pdf]

Supplementary Table S2 : Item rest correlations and item test correlations of every questions of the Children and Adolescents Physical Activity and Sedentary-Questionnaire (CAPAS-Q) for overall sample and subsamples (males and females, children and adolescents)

	Overall		Males		Females		[8-11] years old		[12-18] years old	
**Q**	**IRC**	**ITC**	**IRC**	**ITC**	**IRC**	**ITC**	**IRC**	**ITC**	**IRC**	**ITC**
**Q1**	0.129	0.268	0.203	0.341	0.063	0.200	0.431	0.556	0.039	0.105
**Q2**	0.486	0.592	0.512	0.616	0.445	0.552	0.305	0.446	0.587	0.678
**Q3**	0.262	0.392	0.300	0.430	0.226	0.355	0.207	.0357	0.338	0.462
**Q4**	0.276	0.405	0.221	0.358	0.295	0.418	0.203	0.048	0.481	0.587
**Q5**	0.250	0.381	0.151	0.293	0.300	0.423	0.315	0.455	0.146	0.285
**Q6**	0.374	0.493	0.272	0.405	0.460	0.565	0.434	0.558	0.367	0.448
**Q7**	0.407	0.522	0.238	0.373	0.545	0.638	0.122	0.277	0.546	0.643
**Q8**	0.335	0.459	0.390	0.509	0.312	0.434	0.389	0.520	0.261	0.392
**Q9**	0.553	0.649	0.609	0.697	0.512	0.610	0.416	0.544	0.533	0.632
**Q10**	0.253	0.384	0.315	0.443	0.224	0.354	0.313	0.453	0.150	0.289
**Q11**	0.204	0.339	0.330	0.458	0.206	0.337	0.019	0.178	0.171	0.309
**Q12**	0.281	0.410	0.285	0.416	0.303	0.426	0.116	0.272	0.265	0.396
**Q13**	0.242	0.374	0.256	0.390	0.231	0.359	0.014	0.146	0.250	0.383
**Q14**	0.253	0.384	0.161	0.302	0.330	0.450	0.148	0.302	0.303	0.431
**Q15**	NE	NE	NE	NE	NE	NE	NE	NE	NE	NE
**Q16**	0.223	0.356	0.011	0.157	0.435	0.543	0.341	0.478	0.190	0.327
**Q17**	0.264	0.306	0.284	0.416	0.04	0.089	0.146	0.300	0.036	0.108
**Q18**	0.251	0.383	0.090	0.234	0.379	0.496	0.345	0.482	0.307	0.435
**Q19**	0.134	0.300	0.081	0.262	0.270	0.422	0.048	0.241	0.078	0.249
**Q20**	0.322	0.472	0106	0.286	0.415	0.549	0.354	0.518	0.339	0.488
**Q21**	0.319	0.469	0.389	0.540	0.231	0.387	0.029	0.223	0.502	0.625
**Q22**	0.315	0.465	0.194	0.368	0.432	0.564	0.591	0.710	0.225	0.389
**Q23**	0.468	0.597	0.213	0.385	0.578	0.685	0.348	0.513	0.513	0.639
**Q24**	0.359	0.504	0.060	0.242	0.489	0.612	0.109	0.299	0.481	0.608

**Q25**	0.253	0.410	0.113	0.293	0.333	0.478	0.213	0.395	0.275	0.430
**Q26**	0.269	0.425	0.385	0.537	0.159	0.321	0.205	0.388	0.184	0.348
**Q27**	0.267	0.423	0.157	0.334	0.266	0.418	0.246	0.424	0.171	0.336
**Q28**	0.371	0.514	0.493	0.626	0.294	0.443	0.112	0.302	0.455	0.586
**Q29**	0.437	0.570	0.600	0.711	0.312	0.459	0.427	0.579	0.544	0.660
**Q30**	0.292	0.355	0.225	0.396	0.133	0.296	0.118	0.076	0.119	0.287
**Q31**	0.168	0.332	0.278	0.443	0.161	0.332	0.308	0.478	0.023	0.150

ITC item test correlations ; IRT : Item rest correlations
