# Supplementary material for: Development of the Children and Adolescents Physical Activity and Sedentary Questionnaire (CAPAS-Q): Psychometric Validity and Clinical Interpretation
Source: Int J Environ Res Public Health. 2022 Oct 23;19(21):13782. doi: 10.3390/ijerph192113782 (PMC9655272; doi:10.3390/ijerph192113782)
Supplement: Supplementary file 1 [file ijerph-19-13782-s001.zip › Supplementary Table S3.pdf]

**Supplementary Table S3:** Lin's coefficient of PA and SB dimensions and subdomains derived from the Children and Adolescents Physical Activity and Sedentary-Questionnaire (CAPAS-Q) for overall sample and subsamples (males and females, children and adolescents)

| Dimensions and subdomains | Overall (n = 103) | Males (n = 52) | Females (n = 51) | p-value | [8-11] years old (n = 39) | [12-18] years old (n = 64) | p-value |
|---|---|---|---|---|---|---|---|
| **PA dimension** | 0.693 | 0.586 | 0.750 | 0.193 | 0.723 | 0.666 | 0.667 |
| **PA duration** | 0.756 | 0.657 | 0.833 | 0.075 | 0.914 | 0.659 | 0.003 |
| **PA intensity** | 0.616 | 0.531 | 0.684 | 0.289 | 0.638 | 0.607 | 0.841 |
| **School PA** | 0.675 | 0.452 | 0.794 | 0.010 | 0.560 | 0.701 | 0.363 |
| **Home PA** | 0.602 | 0.483 | 0.690 | 0.165 | 0.470 | 0.625 | 0.389 |
| **Sports and leisure PA** | 0.623 | 0.620 | 0.626 | 0.968 | 0.660 | 0.595 | 0.689 |
| **SB dimension** | 0.684 | 0.552 | 0.781 | 0.076 | 0.357 | 0.815 | 0.004 |
| **SB without screen** | 0.621 | 0.558 | 0.651 | 0.674 | 0.340 | 0.618 | 0.165 |
| **Screen SB** | 0.595 | 0.511 | 0.685 | 0.238 | 0.498 | 0.652 | 0.379 |
| **Consecutive SB time** | 0.725 | 0.650 | 0.821 | 0.099 | 0.730 | 0.723 | 0.952 |
| **School SB** | 0.524 | 0.389 | 0.627 | 0.162 | 0.418 | 0.577 | 0.418 |
| **Home SB** | 0.610 | 0.423 | 0.771 | 0.014 | 0.347 | 0.720 | 0.039 |
| **Transports SB** | 0.535 | 0.375 | 0.734 | 0.019 | 0.276 | 0.551 | 0.200 |
